# The Impact of Lesion-Specific and Sampling-Related Factors on Success of Salivary Gland Fine-Needle Aspiration Cytology

**DOI:** 10.1007/s12105-024-01741-3

**Published:** 2025-01-07

**Authors:** Marcel Mayer, Mohammad Marwan Alfarra, Kathrin Möllenhoff, Marianne Engels, Christoph Arolt, Alexander Quaas, Philipp Wolber, Louis Jansen, Lisa Nachtsheim, Maria Grosheva, Jens Peter Klussmann, Sami Shabli

**Affiliations:** 1 Department of Otorhinolaryngology, Head and Neck Surgery, Medical Faculty, University of Cologne, Cologne, Germany; 2 Institute of Medical Statistics and Computational Biology, University of Cologne, Cologne, Germany; 3 Medical Faculty, Institute of Pathology, University of Cologne, Cologne, Germany

**Keywords:** Fine-needle aspiration cytology, Salivary gland carcinoma, Sonography, Head and neck carcinoma, Cytology, Milan system for reporting salivary gland cytopathology

## Abstract

**Purpose:**

Ultrasound-guided fine-needle aspiration cytology (FNAC) is a widely used diagnostic procedure which facilitates the differentiation of salivary gland lesions. Although the performance of salivary gland FNAC (SG-FNAC) has improved since the introduction of the Milan System for Reporting Salivary Gland Cytopathology (MSRSGC), the range of the reported performance is still wide. Therefore, the aim of this study was to determine lesion- and sampling-related factors that influence the success of SG-FNAC.

**Methods:**

All SG-FNAC cases performed in a tertiary referral hospital between September 1st, 2011, and August 31st, 2022, were retrospectively identified. Demographic, histopathological, lesion-specific, and sampling-related data were retrieved from the clinical charts. Cytopathological reports were categorized according to the MSRSGC. The risk of malignancy (ROM), the performance measures, and factors influencing the success of SG-FNAC were calculated.

**Results:**

Overall, 1289 cases with histopathological follow-up diagnosis (out of 1952 SG-FNACs) were included. The ROM was: non-diagnostic = 23.9%, non-neoplastic = 4.4%, atypia of undetermined significance (AUS) = 34.5%, neoplasm-benign = 1.0%, neoplasm-salivary gland neoplasm of uncertain malignant potential (SUMP) = 15.3%, suspicious for malignancy = 74.1%, malignant = 96.2%. The sensitivity, specificity, accuracy, positive, and negative predictive value for differentiating benign from malignant lesions (excluding lesions categorized as AUS and SUMP) were 87.5%, 97.7%, 96.3%, 85.0%, and 98.1%, respectively. A larger lesion size (OR (95% CI) = 1.21 (1.06–1.39), p = 0.004), a higher number of obtained slides (OR (95% CI) = 1.31 (1.17–1.46), p < 0.001), and the physician performing the FNAC (p = 0.047) were independent predictors for a higher success, while localization of the lesion within the submandibular compared to the parotid gland (OR (95% CI) = 0.38 (0.19–0.77), p = 0.008) was an independent predictor for lower success of SG-FNAC.

**Conclusion:**

This is the largest single-center study evaluating SG-FNAC performance to date. It identified independent lesion-and sampling-related factors influencing the success of SG-FNAC. Knowledge of those can improve performance of the procedure.

**Supplementary Information:**

The online version contains supplementary material available at 10.1007/s12105-024-01741-3.

## Introduction

Neoplasms of the salivary glands are rare showing an incidence of 3.0/100,000 per year [1]. Approximately 20–35% of all salivary gland neoplasms are primary salivary gland carcinomas (SGC) consisting of 21 clinically and biologically markedly distinct entities [1–3]. Additionally, secondary SGC such as metastatic solid tumors and lymphomas can manifest in lymph nodes within the parotid gland [4]. Due to the variety of neoplasms within the salivary glands, a structured and meticulous diagnostic workup is important to determine the correct therapy. Especially, the correct preoperative differentiation between a benign and a malignant tumor is crucial in order to plan the extent of the surgical procedure. Imaging alone has shown to be insufficient for differentiating benign from malignant salivary gland tumors with a sensitivity and specificity of 83% and 85% for computed tomography (CT), 81% and 89% for magnetic resonance imaging (MRI), and 63% and 92% for ultrasonography (US), respectively [5]. Therefore, invasive preoperative diagnostic testing seems necessary. The current European and American guidelines for SGC recommend the routine use of fine-needle aspiration cytology (FNAC) in case of a lesion within one of the major salivary glands [6, 7] due to its simple application and low rate of complications [8]. The Milan System for Reporting Salivary Gland Cytopathology (MSRSGC) was first published in 2018 in order to standardize cytopathological salivary gland tumor reports and to subsequently improve accuracy of FNAC for salivary gland tumors [9]. Although markedly improved after introduction of the MSRSGC, the range of reported performance measures of salivary gland FNAC (SG-FNAC) is still wide between studies with sensitivity rates of 71–93% and specificity rates of 96–99% [10]. The main factors influencing the success of FNAC and thereby leading to these wide ranges of performance measures are still largely unknown. Therefore, this study aimed at analyzing lesion- and sampling-related factors influencing the success of FNAC in the largest single-center series of SG-FNAC cases studied to date.

## Methods

A retrospective clinical chart review was conducted to identify all FNAC cases performed at the Department of Otolaryngology, Head and Neck Surgery at the University Hospital Cologne, Germany, between September 1st, 2011, and August 31st, 2022. Demographic, cytopathological, histopathological, lesion-specific, and sampling-related data including number of cytologic slides, and experience of FNAC-performing physician were retrieved. Only cases with a histopathological follow-up diagnosis were included in the analysis.

In all cases, US was performed using a high-resolution US system with a linear probe (5–12 MHz). US-guided FNAC was performed by 41 otolaryngologists with varying experience levels in SG-FNAC. Cytopathologists were neither involved in sample collection nor performed rapid on-site evaluation. In all cases, a needle (internal standard operating procedure: 24 gauge) attached to an empty plastic syringe with an aspiration device and without local anesthesia was utilized. The patient was in a supine position and the physician who performed the procedure was sitting on the patient’s right side. Direct FNAC slides were prepared by smearing the specimens between two slides. The slides were air-dried and sent to the pathology department. Hematoxylin and eosin (H&E), May-Gruenwald-Giemsa (MGG), and in selected cases Papanicolaou (Pap) staining, were performed. Immunocytochemistry was performed in selected cases with sufficient material and morphologically unclear results. Cytopathological diagnosis was made by five board-certified (cyto-) pathologists with special expertise in salivary gland cytology. All cases were classified according to the MSRSGC. The longest diameter (cm) of the lesion reported in the final pathology report was used for analyses related to the lesion size. The study was conducted in accordance with the Declaration of Helsinki and approved by the Ethics Committee of the University of Cologne (approval code: 24–1328).

## Statistical Analysis

For the descriptive analysis, numerical variables are displayed as means ± standard deviation. Categorical and dichotomous variables are given as frequencies and proportions (%), respectively. For validation of FNAC, the performance measures sensitivity, specificity, the positive predictive value (PPV), the negative predictive value (NPV), and the accuracy were calculated. In accordance with a previous study [10], two settings were defined for calculation of the performance measures. For setting 1, the MSRSGC categories V and VI (suspicious for malignancy and malignant) were the positive index test and the MSRSGC categories II and IVa (non-neoplastic and neoplasm-benign) were the negative test. For setting 2, the MSRSGC categories III, IVb, V, and VI (atypia of undetermined significance (AUS), salivary gland neoplasm of uncertain malignant potential (SUMP), suspicious for malignancy, and malignant) were the positive index test and the MSRSGC categories II and IVa (non-neoplastic and neoplasm-benign) were the negative test. An FNAC was defined as successful, if the specimen was sufficient for a cytologic diagnosis (MSRSGC categories II-VI), whereas it was defined as non-successful in case of a non-diagnostic result (MSRSGC category I). In order to examine the relationship between the binary outcome success of an FNAC and several covariates, univariate logistic regressions were performed. All covariates showing a significant association with success of FNAP in the univariate logistic regression were tested for independent association in a multivariate logistic regression model. For each parameter, the odds ratio (OR) and the corresponding 95% confidence intervals (CI) were calculated. For all statistical analyses SPSS software (IBM SPSS Statistics Version 29.0. Armonk, NY: IBM Corp.) was used and a p-value < 0.05 was considered as significant.

## Results

Overall, 1952 salivary gland FNAC cases were identified. Out of these, 1289 cases (66.0%) with histopathological follow-up diagnoses were included in the further analysis. The majority of these were parotid (92.9%), and 7.1% were submandibular gland lesions, respectively. The mean age of patients was 57.4 (± 15.4) years and 53.1% of patients were male. The mean experience of the FNAC-performing physician was 2.6 (± 1.9) years and a mean of 3.4 (± 1.9) slides per case were obtained (Table 1). The numbers of FNACs performed per physician and the percentage of non-diagnostic FNACs per physician are displayed in Supplementary Table S1.

**Table 1:** Basic characteristics of included patients, lesions, and sampling

Variable	N, SD/(%)
Mean patient age	57.4 ± 15.4
Sex	
Female	604 (46.9%)
Male	685 (53.1%)
Side	
Right	640 (49.7%)
Left	649 (50.3%)
Mean tumor size (cm)	2.5 ± 1.3
Gland	
Parotid gland	1,198 (92.9%)
Submandibular gland	91 (7.1%)
Mean FNAC-performing physician’s experience (years)	2.6 ± 1.9
Mean number of slides per case	3.4 ± 1.9
Mean number of H&E-stained slides	1.7 ± 1.0
Mean number of MGG-stained slides	1.6 ± 1.0
Mean number of Pap-stained slides	0.1 ± 0.2
Immunocytochemistry	
Yes	43 (3.3%)
No	1,246 (96.7%)

*H&E* hematoxylin and eosin staining, MGG May-Gruenwald-Giemsa stain, Pap Papanicolaou stain, SD Standard deviation

**Fig. 1:**
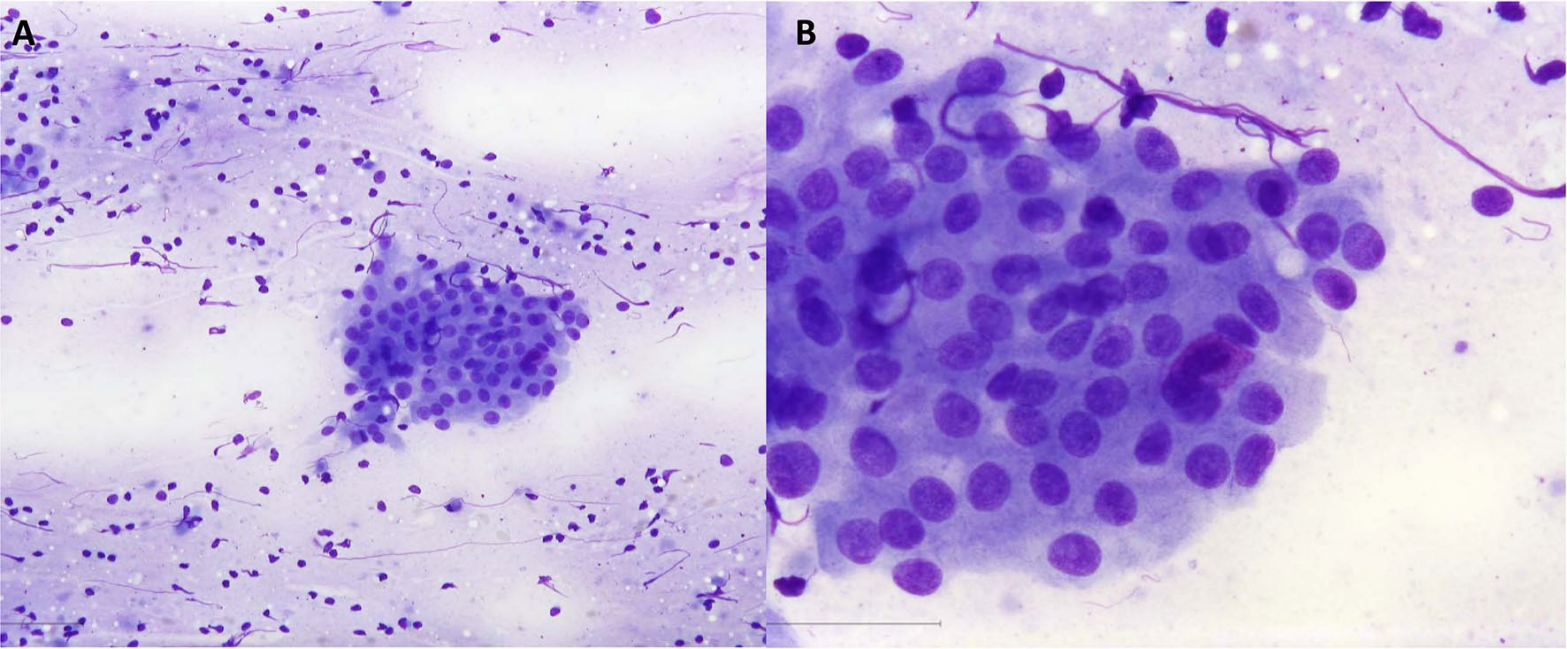
Cytological smear sample of a Warthin’s tumor with a flat sheet of oncocytic epithelium and numerous lymphocytes and mucus in the background. Oncocytes with round nuclei and a smooth nuclear border. Fine chromatin pattern with a discernible nucleolus in some of the nuclei. **A** 20× and **B** 63× . MGG stain

**Fig. 2:**
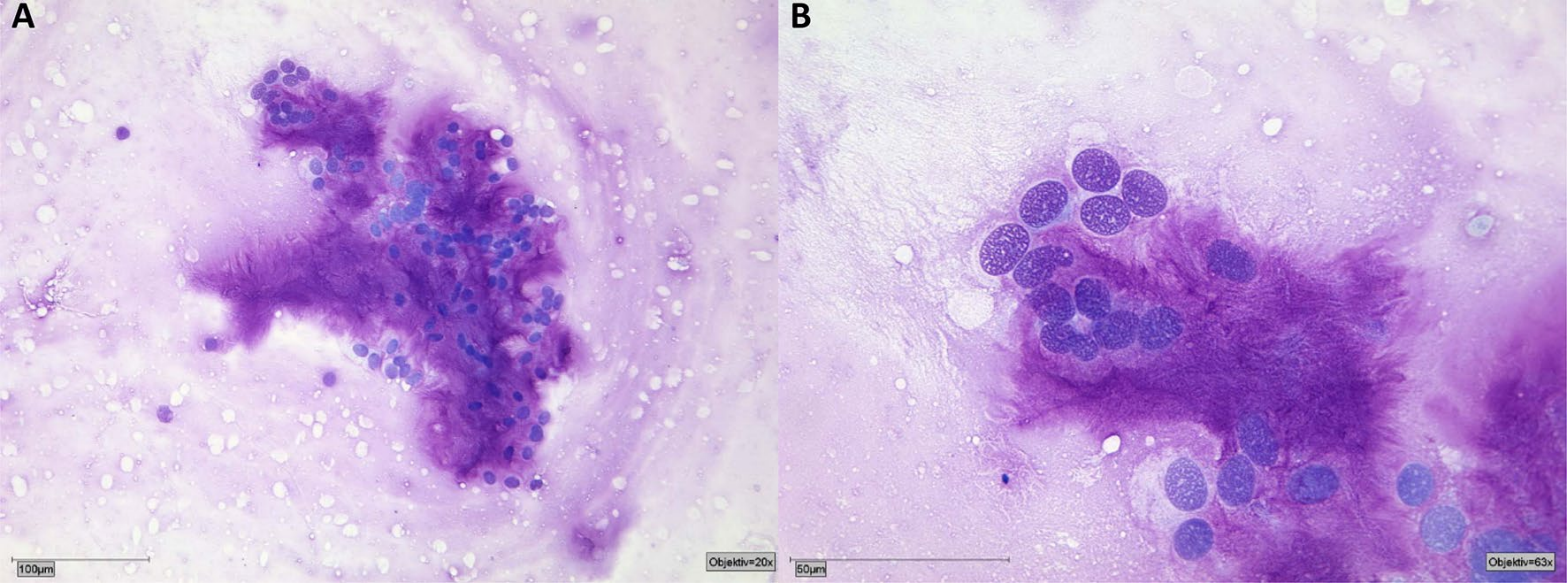
Cytological smear sample of a pleomorphic adenoma with myoepithelial cells embedded in matrix material. Myoepithelial cells with small, round to oval nuclei with a smooth nuclear border and a granular chromatin pattern. The myxoid matrix is stained intensely red to violet in MGG stain and has a fibrillary structure. This type of matrix is typically found in pleomorphic adenoma. **A** 20× and **B** 63x. MGG stain

**Fig. 3:**
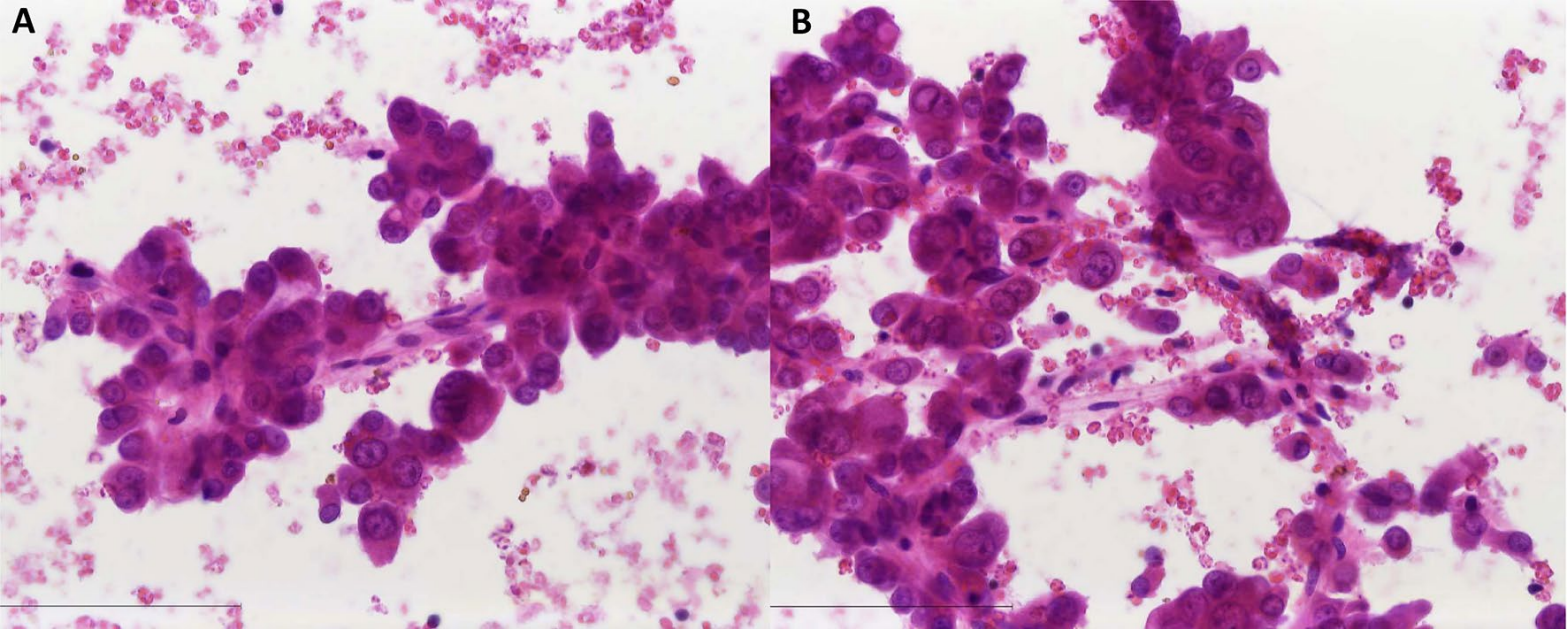
Cytological smear sample of a salivary duct carcinoma showing large tumor cells with large nuclei and prominent nucleoli. The tumor cells are arranged in dense three-dimensional clusters with some papillary fronds. **A** 20× and **B** 63× . H&E stain

Histopathological follow-up revealed 181 (14.0%) non-neoplastic lesions, 879 (68.2%) benign tumors, and 229 (17.8%) malignant tumors. Warthin’s tumor (47.9%) (Fig. 1) was the most common benign tumor, followed by pleomorphic adenoma (44.1%) (Fig. 2), and basal cell adenoma (1.8%). The most frequent malignant tumor was squamous cell carcinoma (SCC) (31.0%), followed by lymphoma (20.1%), mucoepidermoid carcinoma (8.3%), salivary duct carcinoma (5.7%) (Fig. 3), acinic cell carcinoma (5.7%), and metastatic malignant melanoma (5.7%) (Table 2).

**Table 2:** Benign and malignant histopathological diagnoses

	N, (%)
**Benign tumors**	879 (79.3)
Warthin’s tumor	421 (47.9)
Pleomorphic adenoma	388 (44.1)
Basal cell adenoma	16 (1.8)
Oncocytoma	12 (1.4)
Myoepithelioma	9 (1.0)
**Malignant tumors**	229 (20.7)
Squamous cell carcinoma	71 (31.0)
Lymphoma	47 (20.1)
Mucoepidermoid carcinoma	19 (8.3)
Salivary duct carcinoma	13 (5.7)
Acinic cell carcinoma	13 (5.7)
Metastatic malignant melanoma	13 (5.7)
Epithelial-myoepithelial carcinoma	8 (3.5)
Salivary adenocarcinoma, NOS	8 (3.5)
Adenoid cystic carcinoma	7 (3.1)
Basal cell adenocarcinoma	7 (3.1)
Secretory carcinoma	4 (1.7)
Metastatic breast cancer	3 (1.3)
Carcinoma ex pleomorphic adenoma	2 (0.9)
Carcinosarcoma	2 (0.9)
Metastatic merkel cell carcinoma	2 (0.9)
Oncocytic carcinoma	2 (0.9)
Metastatic non-small cell lung cancer	2 (0.9)
Myoepithelial carcinoma	1 (0.4)
Metastatic neuroblastoma	1 (0.4)
Malignant peripheral nerve sheath tumor	1 (0.4)
Langerhans cell histiocytosis	1 (0.4)
Metastatic adenocarcinoma of unknown primary	1 (0.4)
Metastatic renal cell carcinoma	1 (0.4)

*NOS*: Not otherwise specified

MSRSGC IVa (neoplasm-benign) was the most frequent category (38.7%). Risk of neoplasm (RON) was the highest (98.2%) for cases classified as MSRSGC VI (malignant) and the lowest (55.5%) for cases classified as MSRSGC II (non-neoplastic). Risk of malignancy (ROM) was 96.2% for cases classified as MSRSGC VI (malignant) and 1.0% for cases classified as MSRSGC IVa (neoplasm-benign), respectively (Table 3). Among the rare benign tumors, two out of twelve oncocytomas (16.7%) were classified as non-diagnostic, five (41.7%) as non-neoplastic, two (16.7%) as AUS, two (16.7%) as neoplasm-benign, and one (8.3%) as neoplasm-SUMP. For the myoepitheliomas, four out of nine (44.4%) were classified as non-diagnostic, one (11.1%) as non-neoplastic, one (11.1%) as AUS, two (22.2%) as neoplasm-benign, and one (11.1%) as neoplasm-SUMP.

**Table 3:** Diagnostic categories according to the Milan System for Reporting Salivary Gland Cytopathology (MSRSGC)

Category	Final diagnosis			Total n (%)	RON (%)		ROM (%)
	Non-neoplastic	Benign	Malignant				
Non-diagnostic, I	61	178	75	314 (24.2%)	80.6		23.9
Non-neoplastic, II	81	93	8	182 (14.0%)	55.5		4.4
AUS, III	19	55	39	113 (8.7%)	83.2		34.5
Neoplasm-benign, IVa	10	486	5	501 (38.7%)	98.0		1.0
Neoplasm-SUMP, IVb	6	55	11	72 (5.6%)	91.7		15.3
SFM, V	3	11	40	54 (4.2%)	94.4		74.1
Malignant, VI	1	1	51	53 (4.1%)	98.2		96.2
Total	181	879	229	1,289 (100.0%)	86.0		17.8

AUS atypia of undetermined significance, SUMP salivary gland neoplasm of uncertain malignant potential, SFM Suspicious for Malignancy, RON risk of neoplasm, ROM risk of malignancy

For distinguishing malignant from benign tumors excluding tumors classified as AUS and SUMP (Setting 1), sensitivity, specificity, accuracy, PPV, and NPV were as follows: 87.5%, 97.7%, 96.3%, 85.0%, and 98.1%. Notably, B-cell lymphomas accounted for 4 out of 13 false-negative FNAC results. For distinguishing malignant from benign tumors including tumors classified as AUS and SUMP (Setting 2), sensitivity, specificity, accuracy, PPV, and NPV were as follows: 96.1%, 81.6%, 83.2%, 48.3%, and 98.1% (Table 4).

**Table 4:** Diagnostic efficacy of the Milan System for Reporting Salivary Gland Cytopathology (MSRSGC) for different settings

Setting	Sensitivity, %	Specificity, %	Accuracy, %	PPV, %	NPV, %
Setting 1	87.5	97.7	96.3	85.0	98.1
Setting 2	91.6	81.6	83.2	48.3	98.1

Setting 1: Suspicious for malignancy (SFM) and malignant (M) were the positive index test, whereas non-neoplastic (NN) and neoplasm-benign (NB) were the negative test

Setting 2: Atypia of undetermined significance (AUS), SUMP, SFM, and M were the positive index test, whereas NN and NB were the negative test

The false-positive cases within the malignant and suspicious categories were as follows: among the malignant category (MSRSGC VI), there were two false-positive cases. One chronic sialadenitis within the submandibular gland was misdiagnosed as indolent non-Hodgkin lymphoma and one pleomorphic adenoma within the parotid gland was misdiagnosed as solid malignant tumor. Additionally, fourteen false-positive diagnoses were made in the SFM category (MSRSGC V). The final diagnoses were five Warthin’s tumors, four pleomorphic adenomas, two basal cell adenomas, two cases with chronic sialadenitis, and one cyst.

Further, five false-negative diagnoses were made in the neoplasm-benign (MSRSGC IVa) category. One acinic cell carcinoma and one mucoepidermoid carcinoma were misdiagnosed as Warthin’s tumors, and one salivary adenocarcinoma not otherwise specified, one salivary duct carcinoma, and one mucoepidermoid carcinoma were misdiagnosed as pleomorphic adenomas.

Table 5 and Supplementary Table S2 display the results from the univariate logistic regression. The estimated coefficients show that a larger tumor size significantly increased the probability of a successful FNAC with a higher success rate of 22% per additional cm of size (OR (95% CI) = 1.22 (1.07–1.39), p = 0.003). For the submandibular gland, it was less likely to obtain a successful FNAC compared to the parotid gland (OR (95% CI) = 0.54 (0.34–0.84), p = 0.007). While the side of the lesion (right vs. left) did not show a significant association with the probability of success (OR (95% CI) = 0.92 (0.71–1.19), p = 0.525), the number of slides was positively associated with a successful FNAC. More precisely, each additional slide led to an increased probability of success of 31% (OR (95% CI) = 1.31 (1.20–1.43), p < 0.001). Further, the individual physician who performed the FNAC had a significant influence on the success of the procedure (p = 0.009). Notably, the individual physician’s experience was not associated with the success of the FNAC (OR (95% CI) = 0.96 (0.89–1.03), p = 0.219). Lastly, the pathologist who assessed the slides was not predictive for the success of the FNAC (p = 0.996). The multivariate logistic regression model confirmed the independent influence of the lesion-related variables tumor size (OR (95% CI) = 1.21 (1.06–1.39), p = 0.004)) and lesion in the submandibular compared to the parotid gland (OR (95% CI) = 0.38, (0.19–0.77), p = 0.008) as well as the sampling-related variables number of slides (OR (95% CI) = 1.31 (1.17–1.46), p < 0.001) and FNAC-performing physician on success of the procedure (p = 0.047) (Table 6, Supplementary Table S3).

**Table 5:** Univariate logistic regression for influence of lesion-and sampling-related variables on success of fine-needle aspiration cytology

Variable	B (SE)	OR (95% CI)		p-value
Tumor size	0.20 (0.07)	1.22 (1.07–1.39)		**0.003**
Gland				
Parotid gland		1.00		
Submandibular gland	− 0.62 (0.23)	0.54 (0.34–0.84)		**0.007**
Side				
Left		1.00		
Right	− 0.08 (0.13)	0.92 (0.71–1.19)		0.525
Number of slides	0.27 (0.05)	1.31 (1.20–1.43)		** < 0.001**
FNAC-performing physician’s experience	− 0.05 (0.37)	0.96 (0.89–1.03)		0.219
FNAC-performing physician				**0.009**
Physician 1	− 0.43 (0.46)	0.65 (0.26–1.62)		0.354
…				
Physician 20	− 0.91 (0.33)	0.40 (0.21–0.78)		**0.007**
…				
Physician 30	− 1.44 (0.63)	0.24 (0.08–0.68)		**0.007**
…				
Physician 36	− 2.00 (1.05)	0.14 (0.04–0.47)		**0.001**
…				
Physician 41	− 0.46 (0.39)	0.63 (0.32–1.22)		0.170
FNAC-assessing pathologist				0.996
Pathologist 1	− 0.39 (0.79)	0.68 (0.15–3.18)		0.625
Pathologist 2	− 0.33 (0.80)	0.72 (0.15–3.43)		0.676
Pathologist 3	**	**		0.999
Pathologist 4	− 0.41 (0.79)	0.67 (0.14–3.16)		0.609
Pathologist 5	**	**		0.999

B: Logistic regression coefficient, SE Standard error, OR Odds ratio, CI confidence interval, FNAC fine-needle aspiration cytology, significant results in bold letters

**not applicable due to low number of assessed cases, detailed data for FNAC-performing physicians in Supplementary Table S2

**Table 6:** Multivariate logistic regression for influence of lesion- and sampling-related variables on success of fine-needle aspiration cytology

Variable	B (SE)	OR (95% CI)	p-value
Tumor size (cm)	0.19 (0.07)	1.21 (1.06–1.39)	**0.004**
Gland			
Parotid gland		1.00	
Submandibular gland	− 0.98 (0.37)	0.38 (0.19–0.77)	**0.008**
Number of slides	0.27 (0.06)	1.31 (1.17–1.46)	** < 0.001**
FNAC-performing Physician			**0.047**
Physician 1	− 0.52 (0.54)	0.60 (0.21–1.72)	0.339
…			
Physician 20	− 1.12 (0.37)	0.33 (0.16–0.67)	**0.002**
…			
Physician 30	− 1.42 (0.57)	0.24 (0.08–0.74)	**0.012**
…			
Physician 36	− 2.27 (0.74)	0.10 (0.02–0.44)	**0.002**
…			
Physician 41	− 1.80 (0.88)	0.17 (0.03–0.92)	0.178

B: Logistic regression coefficient, SE Standard error, OR Odds ratio, CI confidence interval, significant results in bold letters, detailed data for FNAC-performing physicians in Supplementary Table S3

## Discussion

This is the first study analyzing various lesion- and sampling- related factors for their influence on the success of SG-FNAC in the largest single-center study conducted for SG-FNAC to date.

Among the 1289 cases, the mean patient age (57.4 ± 15.4) was slightly higher than the mean age of 54.4 years reported in the existing literature [10]. In accordance with prior studies [10], male patients were predominant compared to female ones (male-to-female ratio = 1.1:1). The mean size of lesions (2.5 ± 1.3 cm) was slightly higher than the mean lesion size of around 2.3 cm, which had been reported in a previously published study [11].

The most common benign entity in this series was Warthin’s tumor accounting for 47.9% of all benign tumors, which is congruent with recent data suggesting that Warthin’s tumor has been the most frequent benign parotid gland tumor found in surgical series within the last two decades [12, 13]. Further, SCC was with 31.0% the most frequent malignant entity which is confirming previous studies showing an increasing incidence of mostly metastatic cutaneous SCC to the parotid gland [4, 14]. Notably, 20.1% of all malignant tumors in this study were lymphomas, which poses a markedly higher frequency than previously reported in studies evaluating SG-FNAC showing that 6.3–12.4% of all malignant neoplasms were lymphomas [15–17].

The ROM for MSRSGC category I (non-diagnostic) was 23.9% in this series, which is higher than recommended in the second edition of the MSRSGC (15%) [18] and in a large meta-analysis by Wang et al. containing 7,168 SG-FNAC cases (11.4%) [10]. This finding is most likely due to the fact that SG-FNAC is performed by all otolaryngologists in our department (mean SG-FNAC experience: 2.6 years) resulting in a frequency of 24.2% of cases being classified as non-diagnostic. This setting differs from many other institutions, where SG-FNAC is performed by a limited number of specialized physicians and therefore shows a markedly lower rate of non-diagnostic results. One example is a study by Kim et al., where FNAC performed by one radiologist with an experience of 6 years in SG-FNAC led to a rate of 3.2% of SG-FNACs being inadequate [11]. Furthermore, the absence of rapid on-site evaluation to determine the adequacy of the specimen would most likely have increased the percentage of non-diagnostic cases. On the other hand, among 501 cases classified as MRSRSGC category IVa (neoplasm-benign), the ROM was only 1.0%, which is markedly lower than in the meta-analysis by Wang et al. (2.8%) [10] and emphasizes the high reliability of SG-FNAC in case of an adequate sample and a highly specialized cytologist. Moreover, the ROM was as low as 4.4% for MSRSGC category II (non-neoplastic) in our study, which is likewise lower than reported in the aforementioned meta-analysis (10.9%) [10] and recommended in the second edition of the MSRSGC (11%) [18].

For differentiation between benign and malignant lesions when only including definite FNAC diagnoses (setting 1), the sensitivity and specificity were 87.5% and 97.7% in this study, which is in accordance with a pooled sensitivity and specificity of 88.0% and 98.5%, respectively, shown in the meta-analysis by Wang et al. for the same setting [10]. It must be mentioned that 4 out of 13 false-negative results in this setting were found in B-cell lymphomas, which had been shown to have a particularly high risk of false-negative results [10, 19, 20]. Therefore, it can be assumed that a lower frequency of lymphomas (as reported in most other studies evaluating the performance of SG-FNAC), would have led to a higher sensitivity in this setting. After adding SUMP and AUS to the analysis (setting 2), the sensitivity increased slightly to 91.6%, but the specificity decreased markedly to 81.6%, as expected. This result confirms the usefulness of the MSRSGC, which provides the cytopathologist with the possibility to classify SG-FNACs as SUMP or AUS and therefore prevents providing potentially misleading definite diagnoses.

The results of this study showed a significant influence of various lesion- and sampling-related factors on success of SG-FNAC. First, a larger lesion size was an independent predictor for adequacy of the sample. In more detail, the success rate of FNAC was increased by 22% with each additional cm of lesion size. In the only study which had investigated factors influencing SG-FNAC outcomes to date, the tumor size was not associated with a higher success rate [11], but another study has shown that sensitivity, specificity, and accuracy were higher for parotid lesions measuring 2.1–4.0 cm compared to those smaller than 2 cm [21]. It has to be mentioned that in both studies the independence of this factor has not been evaluated in a multivariate analysis. Further, the present study revealed that success of FNAC was significantly lower for lesions within the submandibular compared to the parotid gland. The previously mentioned study by Kim et al. could not show an association between the location of the lesion and FNAC outcome, which is most likely due to the markedly lower number of cases included in that study [11]. A lower success of FNAC for submandibular compared to parotid lesions may be explained by the worse accessibility of the submandibular gland due to its location in the submandibular triangle and emphasizes the need for a particularly meticulous FNAC procedure in these cases. Although the FNAC-performing physician was sitting on the right patient’s side in all cases, the side of the lesion was not a predictive factor. Consequently, it seems that this setting is sufficient for lesions on both patient sides. A higher number of slides was a further independent predictor for a successful SG-FNAC (31% higher chance of successful FNAC per each additional slide). It can be assumed that the number of slides is a surrogate parameter for the amount of material obtained during the procedure. Therefore, it may be necessary to perform a second puncture in case of insufficient material in order to increase the likelihood of an adequate sample. Lastly, the study showed that the individual FNAC-performing physician had a significant influence on success of SG-FNAC. The influence of the physician could not be explained by the experience level, which is in contrast to a prior study revealing that the experience of the physician was a significant predictor for a diagnostic FNAC result [22]. Both findings highlight the relevance of the technique used during the procedure. It may be helpful to identify physicians with the highest success rates within each department and to adopt technical aspects relevant to the FNAC procedure.

This study has several limitations. First, the typical limitations of retrospective data collection have to be considered when interpreting the results. Further, although the internal standard operating procedure recommends a 24-gauge needle, the needle size was unknown for each particular procedure and can therefore not be excluded as a confounder. On the other hand, numerous previous studies have shown that the needle size was not associated with the outcome of FNAC [11, 23–25]. Lastly, the exact number of needle passes was unknown for each particular procedure. Although the internal standard operating procedure recommends only one pass, a higher number of needle passes in selected cases cannot be excluded. It has to be mentioned that for FNAC of thyroid nodules there is contradictory evidence regarding the additional value of more than one pass for improvement of success of the procedure [22, 26]. Therefore, further studies should address this research question for SG-FNAC.

Despite the limitations, this study represents the largest single-center study evaluating the performance of SG-FNAC. Most importantly, it is the first study revealing independent lesion- and sampling-related predictors influencing the success rate of SG-FNAC. According to these findings, the success rate of SG-FNAC may be markedly improved by generating as many slides as possible, being particularly precise in cases of smaller lesions and lesions within the submandibular gland, and by identifying physicians with a particularly high SG-FNAC success rate.

## Supplementary Information

Below is the link to the electronic supplementary material.

Supplementary file1 (DOCX 41 KB)

## Data Availability

The data presented in this study are available on request from the corresponding author. The data are not publicly available due to privacy restrictions.
