# Supplementary material for: The Impact of Lesion-Specific and Sampling-Related Factors on Success of Salivary Gland Fine-Needle Aspiration Cytology
Source: Head Neck Pathol. 2025 Jan 7;19(1):1. doi: 10.1007/s12105-024-01741-3 (PMC11707220; doi:10.1007/s12105-024-01741-3)
Supplement: Supplementary file 1 — Supplementary file1 (DOCX 41 KB) [file 12105_2024_1741_MOESM1_ESM.docx]

**Supplementary Table S1. Number of FNACs and rate of non-diagnostic FNACs per physician**

| **Physician ID** | **Number of FNACs (n)** | **Non-diagnostic rate (%)** |
| --- | --- | --- |
| 1 | 25 | 32.0 |
| 2 | 23 | 21.7 |
| 3 | 8 | 12.5 |
| 4 | 65 | 18.5 |
| 5 | 29 | 27.6 |
| 6 | 18 | 27.8 |
| 7 | 30 | 40.0 |
| 8 | 15 | 6.7 |
| 9 | 55 | 32.7 |
| 10 | 8 | 12.5 |
| 11 | 10 | 10.0 |
| 12 | 38 | 21.1 |
| 13 | 15 | 13.3 |
| 14 | 45 | 22.2 |
| 15 | 24 | 20.8 |
| 16 | 22 | 13.6 |
| 17 | 19 | 15.8 |
| 18 | 18 | 38.9 |
| 19 | 32 | 28.1 |
| 20 | 46 | 23.9 |
| 21 | 24 | 25.0 |
| 22 | 14 | 14.3 |
| 23 | 26 | 3.8 |
| 24 | 44 | 22.7 |
| 25 | 18 | 5.6 |
| 26 | 57 | 24.6 |
| 27 | 51 | 43.1 |
| 28 | 14 | 35.7 |
| 29 | 5 | 20.0 |
| 30 | 8 | 44.4 |
| 31 | 16 | 18.8 |
| 32 | 43 | 16.3 |
| 33 | 54 | 16.7 |
| 34 | 22 | 22.7 |
| 35 | 11 | 45.5 |
| 36 | 18 | 56.3 |
| 37 | 64 | 23.4 |
| 38 | 46 | 15.2 |
| 39 | 6 | 33.3 |
| 40 | 7 | 28.6 |
| 41 | 13 | 69.2 |

*FNAC: Fine-needle aspiration cytology*

**Supplementary Table S2. Univariate logistic regression results for influence of FNAC-performing physicians on success of FNAC**

|  | | B | S.E. | Wald | P-value | OR | 95% C.I. for OR | |
| --- | --- | --- | --- | --- | --- | --- | --- | --- |
|  |  |  |  |  |  |  | Lower | Upper |
|  | Tumor size | ,198 | ,066 | 9,021 | ,003 | 1,219 | 1,071 | 1,386 |
|  | Parotid gland |  |  |  |  | 1,000 |  |  |
|  | Submandibular gland | -,619 | ,228 | 7,337 | ,007 | 0,539 | ,344 | ,843 |
|  | Left side |  |  |  |  | 1,000 |  |  |
|  | Right side | -,083 | 0,130 | ,405 | ,525 | ,921 | ,714 | 1,188 |
|  | Number of slides | ,267 | ,045 | 34,936 | < ,001 | 1,306 | 1,195 | 1,427 |
|  | Physician |  |  |  | ,009 |  |  |  |
|  | Physician 1 | -,430 | ,464 | ,859 | ,354 | ,650 | ,262 | 1,616 |
|  | Physician 2 | ,762 | 1,084 | ,494 | ,482 | 2,142 | ,256 | 17,920 |
|  | Physician 3 | 1,013 | 1,069 | ,898 | ,343 | 2,754 | ,339 | 22,384 |
|  | Physician 4 | ,137 | ,436 | ,099 | ,753 | 1,147 | ,488 | 2,697 |
|  | Physician 5 | ,688 | ,780 | ,776 | ,378 | 1,989 | ,431 | 9,178 |
|  | Physician 6 | ,068 | ,401 | ,029 | ,864 | 1,071 | ,488 | 2,348 |
|  | Physician 7 | ,151 | ,533 | ,080 | ,777 | 1,163 | ,409 | 3,307 |
|  | Physician 8 | ,662 | ,646 | 1,047 | ,306 | 1,938 | ,546 | 6,879 |
|  | Physician 9 | ,490 | ,654 | ,561 | ,454 | 1,632 | ,453 | 5,880 |
|  | Physician 10 | -,732 | ,515 | 2,019 | ,155 | ,481 | ,175 | 1,320 |
|  | Physician 11 | -,246 | ,432 | ,325 | ,569 | ,782 | ,335 | 1,823 |
|  | Physician 12 | ,097 | ,536 | ,033 | ,857 | 1,101 | ,385 | 3,150 |
|  | Physician 13 | -,027 | ,389 | ,005 | ,945 | ,974 | ,454 | 2,087 |
|  | Physician 14 | -,086 | ,504 | ,029 | ,865 | ,918 | ,342 | 2,465 |
|  | Physician 15 | ,607 | ,784 | ,600 | ,439 | 1,836 | ,395 | 8,540 |
|  | Physician 16 | 2,035 | 1,035 | 3,862 | ,049 | 7,649 | 1,005 | 58,193 |
|  | Physician 17 | ,040 | ,402 | ,010 | ,922 | 1,040 | ,474 | 2,286 |
|  | Physician 18 | 1,649 | 1,044 | 2,493 | ,114 | 5,201 | ,672 | 40,280 |
|  | Physician 19 | -,062 | ,356 | ,031 | ,861 | ,940 | ,468 | 1,887 |
|  | Physician 20 | -,908 | ,334 | 7,375 | ,007 | ,403 | ,209 | ,777 |
|  | Physician 21 | -,596 | ,586 | 1,037 | ,308 | ,551 | ,175 | 1,736 |
|  | Physician 22 | ,202 | 1,132 | ,032 | ,858 | 1,224 | ,133 | 11,258 |
|  | Physician 23 | ,762 | 1,084 | ,494 | ,482 | 2,142 | ,256 | 17,920 |
|  | Physician 24 | -,961 | ,507 | 3,596 | ,058 | ,382 | ,142 | 1,033 |
|  | Physician 25 | ,282 | ,665 | ,180 | ,671 | 1,326 | ,360 | 4,881 |
|  | Physician 26 | ,453 | ,450 | 1,015 | ,314 | 1,574 | ,651 | 3,801 |
|  | Physician 27 | ,425 | ,406 | 1,094 | ,296 | 1,530 | ,690 | 3,393 |
|  | Physician 28 | ,040 | ,539 | ,005 | ,942 | 1,040 | ,362 | 2,993 |
|  | Physician 29 | -1,002 | ,631 | 2,519 | ,112 | ,367 | ,107 | 1,265 |
|  | Physician 30 | -1,436 | ,535 | 7,210 | ,007 | ,238 | ,083 | ,679 |
|  | Physician 31 | ,000 | ,345 | ,000 | ,999 | 1,000 | ,508 | 1,965 |
|  | Physician 32 | ,533 | ,448 | 1,420 | ,233 | 1,705 | ,709 | 4,099 |
|  | Physician 33 | -,491 | ,884 | ,308 | ,579 | ,612 | ,108 | 3,462 |
|  | Physician 34 | ,301 | ,366 | ,676 | ,411 | 1,351 | ,659 | 2,770 |
|  | Physician 35 | -,268 | ,855 | ,098 | ,754 | ,765 | ,143 | 4,091 |
|  | Physician 36 | -1,995 | ,627 | 10,130 | ,001 | ,136 | ,040 | ,465 |
|  | Physician 37 | -,219 | ,452 | ,235 | ,628 | ,803 | ,331 | 1,949 |
|  | Physician 38 | -,229 | ,556 | ,169 | ,681 | ,796 | ,268 | 2,364 |
|  | Physician 39 | -,779 | ,413 | 3,552 | ,059 | ,459 | ,204 | 1,032 |
|  | Physician 40 | 1,455 | 1,050 | 1,918 | ,166 | 4,284 | ,547 | 33,565 |
|  | Physician 41 | -,464 | ,338 | 1,879 | ,170 | ,629 | ,324 | 1,221 |

*B: Logistic regression coefficient; SE: Standard error; OR: Odds ratio; CI: confidence interval*

**Supplementary Table S3. Multivariate logistic regression results for influence of FNAC-performing physicians on success of FNAC**

|  | | B | S.E. | Wald | P-value | OR | 95% C.I. for OR | |
| --- | --- | --- | --- | --- | --- | --- | --- | --- |
|  |  |  |  |  |  |  | Lower | Upper |
|  | Tumor size | ,194 | ,068 | 8,083 | ,004 | 1,214 | 1,062 | 1,388 |
|  | Parotid gland |  |  |  |  |  |  |  |
|  | Submandibular gland | -,975 | ,365 | 7,145 | ,008 | ,377 | ,185 | ,771 |
|  | Number of slides | ,267 | ,058 | 21,306 | <,001 | 1,306 | 1,166 | 1,462 |
|  | Physician |  |  |  | ,047 |  |  |  |
|  | Physician 1 | -,517 | ,541 | ,913 | ,339 | ,596 | ,206 | 1,723 |
|  | Physician 2 | ,825 | 1,113 | ,549 | ,459 | 2,282 | ,258 | 20,214 |
|  | Physician 3 | ,833 | 1,095 | ,578 | ,447 | 2,299 | ,269 | 19,659 |
|  | Physician 4 | -,259 | ,481 | ,290 | ,590 | ,772 | ,300 | 1,983 |
|  | Physician 5 | ,055 | ,850 | ,004 | ,948 | 1,057 | ,200 | 5,593 |
|  | Physician 6 | -,246 | ,455 | ,292 | ,589 | ,782 | ,320 | 1,908 |
|  | Physician 7 | ,323 | ,661 | ,239 | ,625 | 1,382 | ,378 | 5,048 |
|  | Physician 8 | ,527 | ,792 | ,443 | ,506 | 1,694 | ,359 | 8,002 |
|  | Physician 9 | ,931 | ,795 | 1,372 | ,241 | 2,538 | ,534 | 12,061 |
|  | Physician 10 | -,608 | ,562 | 1,171 | ,279 | ,545 | ,181 | 1,637 |
|  | Physician 11 | -,533 | ,472 | 1,273 | ,259 | ,587 | ,233 | 1,481 |
|  | Physician 12 | -,367 | ,614 | ,358 | ,550 | ,693 | ,208 | 2,307 |
|  | Physician 13 | -,264 | ,432 | ,374 | ,541 | ,768 | ,329 | 1,790 |
|  | Physician 14 | ,012 | ,554 | ,000 | ,982 | 1,012 | ,342 | 2,998 |
|  | Physician 15 | ,554 | 1,097 | ,255 | ,613 | 1,740 | ,203 | 14,946 |
|  | Physician 16 | 1,359 | 1,048 | 1,682 | ,195 | 3,893 | ,499 | 30,374 |
|  | Physician 17 | -,395 | ,461 | ,732 | ,392 | ,674 | ,273 | 1,665 |
|  | Physician 18 | 1,300 | 1,070 | 1,477 | ,224 | 3,670 | ,451 | 29,860 |
|  | Physician 19 | -,286 | ,401 | ,510 | ,475 | ,751 | ,343 | 1,647 |
|  | Physician 20 | -1,123 | ,369 | 9,243 | ,002 | ,325 | ,158 | ,671 |
|  | Physician 21 | -,254 | ,725 | ,123 | ,726 | ,775 | ,187 | 3,215 |
|  | Physician 22 | ,409 | 1,139 | ,129 | ,720 | 1,505 | ,161 | 14,040 |
|  | Physician 23 | ,203 | 1,143 | ,032 | ,859 | 1,225 | ,130 | 11,507 |
|  | Physician 24 | -,982 | ,581 | 2,853 | ,091 | ,375 | ,120 | 1,171 |
|  | Physician 25 | -,616 | ,831 | ,550 | ,458 | ,540 | ,106 | 2,752 |
|  | Physician 26 | ,733 | ,574 | 1,628 | ,202 | 2,081 | ,675 | 6,415 |
|  | Physician 27 | ,316 | ,442 | ,510 | ,475 | 1,371 | ,577 | 3,261 |
|  | Physician 28 | -,152 | ,634 | ,058 | ,810 | ,859 | ,248 | 2,976 |
|  | Physician 29 | -,751 | ,711 | 1,117 | ,290 | ,472 | ,117 | 1,900 |
|  | Physician 30 | -1,415 | ,566 | 6,250 | ,012 | ,243 | ,080 | ,737 |
|  | Physician 31 | -,205 | ,406 | ,255 | ,613 | ,815 | ,368 | 1,804 |
|  | Physician 32 | ,642 | ,579 | 1,228 | ,268 | 1,900 | ,610 | 5,917 |
|  | Physician 33 | ,227 | 1,167 | ,038 | ,846 | 1,254 | ,127 | 12,351 |
|  | Physician 34 | ,871 | ,490 | 3,156 | ,076 | 2,388 | ,914 | 6,240 |
|  | Physician 35 | -,116 | ,901 | ,017 | ,898 | ,891 | ,152 | 5,209 |
|  | Physician 36 | -2,266 | ,743 | 9,314 | ,002 | ,104 | ,024 | ,444 |
|  | Physician 37 | -,592 | ,510 | 1,344 | ,246 | ,553 | ,203 | 1,505 |
|  | Physician 38 | -,017 | ,833 | ,000 | ,984 | ,983 | ,192 | 5,035 |
|  | Physician 39 | ,487 | ,605 | ,649 | ,420 | 1,628 | ,498 | 5,325 |
|  | Physician 40 | 1,220 | 1,071 | 1,298 | ,255 | 3,387 | ,415 | 27,642 |
|  | Physician 41 | -,523 | ,387 | 1,829 | ,176 | ,593 | ,278 | 1,265 |

*B: Logistic regression coefficient; SE: Standard error; OR: Odds ratio; CI: confidence interval*
